# Editorial: Bacterial Mechanisms of Antibiotic Resistance: A Structural Perspective

**DOI:** 10.3389/fmolb.2019.00071

**Published:** 2019-08-14

**Authors:** Graeme L. Conn, Vassiliy N. Bavro, Christopher Davies

**Affiliations:** ^1^Department of Biochemistry, Emory University School of Medicine, Atlanta, GA, United States; ^2^School of Biological Sciences, University of Essex, Colchester, United Kingdom; ^3^Department of Biochemistry and Molecular Biology, Medical University of South Carolina, Charleston, SC, United States

**Keywords:** antibiotic resistance, antibiotic inactivation, multi-drug-resistance, cell wall alteration, efflux pumps, ribosome, bacteria

Antibiotic-resistant bacteria are responsible for millions of hard-to-treat infections annually. Since antibiotic “miracle drugs” were first introduced into clinical use, resistance has closely followed; more recently, this problem has been greatly exacerbated by their extensive use in medicine and agriculture, combined with the remarkable ability of bacterial populations to rapidly evolve and exchange genetic material. The rise of multidrug resistance (MDR), coupled with declining availability of newly approved or in-development treatments, threatens to fundamentally alter our ability to treat infections. Whether the most pessimistic predictions of a future “post-antibiotic era” become reality over the coming decades will depend on actions taken in the present. This Research Topic collects together articles that highlight the recent contributions of structural biology and related approaches to our understanding of antibiotic resistance and adaptions used by bacteria against drugs that target key cellular structures, complexes or pathways, as well as drug development efforts to counter these resistance mechanisms. New insights from such approaches are likely to be critical in future efforts to develop strategies to overcome existing resistance mechanisms and to identify targets for novel antibiotic development.

## Efflux Pumps and Transporters

The tripartite assemblies built around the Resistance-Nodulation-Division (RND) family of proton-powered secondary transporters play a prominent MDR role in Gram-negative bacteria. While RND assemblies have been a focus of a recent dedicated Frontiers Research Topic (Vargiu et al., 2016), their structural biology is one of the most rapidly advancing directions within the MDR field and two important experimental studies are reported within this collection.

First, Zwama et al. use X-ray crystallography to dissect the role of the so-called hoisting loop, located at the border of transmembrane helix 8 and the PC2 subdomain, in the prototypical RND family member AcrB. This study demonstrates how the random coil-to-α-helix transition of this loop leads to opening and closing of the drug-channel entrance. Crucially, this work elucidates one of the last remaining problematic areas regarding the functional RND-pump cycle, namely the energy transduction and conformational coupling between remote regions of the RND-transporters.

Another persistent question within the RND field is the structural basis for the apparent broad-substrate specificity provided by these pumps. Ramaswamy et al. address this using molecular dynamics simulations of the centrally important RND-transporters MexB and MexY from *Pseudomonas aeruginosa*. This study characterizes the potential binding pockets of these transporters and their substrates which, by innovative use of electrostatic complementarity analysis, allows the authors to reveal key differences between these transporters. Importantly, this first comparative study of the major *P. aeruginosa* transporters suggests that the deep binding pocket of the tight conformer plays a central role in substrate selectivity.

While the role of RND transporters in efflux and tripartite assemblies has been extensively studied, until recently, much less was known about the structural organization of the ABC-transporter family members participating in tripartite assemblies. A wide-ranging review by Greene et al. synthesizes the recent advances in structure and function of the MacB-family of ABC-transporters, which form unique tripartite assemblies with a role in macrolide efflux and protein export. The authors provide a tantalizing novel model of functional mechanotransmission and discuss the links to homologous tripartite systems from other pathogenic bacteria, which similarly export protein-like signaling molecules, virulence factors, and siderophores.

Genetic regulation of efflux pumps is a key mechanism of resistance, with their associated transcriptional regulators emerging as promising therapeutic targets, and yet, this remains one of least well-understood areas in MDR. Bridging this gap, Issa et al., provide a comprehensive review of the recent progress in the structural biology of regulator families in *P. aeruginosa*, including the one-component system regulators of the TetR, LysR, MarR, AraC families, and two-component systems (TCS) families of regulators. In a related work, Milton et al., combine molecular modeling with biochemical and cellular studies to propose a potential mechanism of interaction between TCS response regulators and 2-aminoimidazole compounds which can inhibit bacterial biofilm formation, disperse preformed biofilms, and re-sensitize MDR bacteria to antibiotics. This study focuses on two important pathogens, *Acinetobacter baumannii* and *Francisella tularensis*, and provides promising new insights into this potential new therapeutic avenue.

## Cell Wall Alterations

The complex role played by the bacterial cell-envelope structure, and particularly the lipid A (endotoxin) component of lipopolysaccharide (LPS) outer membrane layer, in modulating the bacterial susceptibility to host antimicrobials such as cationic antimicrobial peptides, is subject to an in-depth review by Kahler et al.. The role of LPS in bacterial pathogenesis and immunological evasion has recently been the focus of increased attention and this work provides a timely summary of the knowledge of the effects of phosphoethanolamine decoration of lipid A in pathogenic *Neisseria* strains and the potential of targeting the EptA-enzyme responsible for therapeutic purposes.

Another way bacteria protect themselves against external agents is by altering the peptidoglycan (PG) cell wall and a well-known example is replacing the d-Ala moiety of PG to d-lactate to confer vancomycin resistance in enterococci. In their review article, Yadav et al. explain how various chemical modifications to PG help defend bacteria against host-generated antimicrobials, such as lysozyme and other hydrolytic enzymes, as well as antibiotics. Such knowledge can help guide new therapeutic approaches that weaken the bacterial cell wall and increase susceptibility to existing antibiotics.

## Ribosome-Targeting Antibiotics and Resistance Mechanisms

Ribosomes are the essential RNA-protein complexes responsible for protein synthesis in all cells. However, unique aspects of the bacterial ribosome allow for specific antibiotics that interfere with every aspect of ribosome function. These chemically diverse drugs have been a major component of our clinical arsenal for many decades and three articles here focus on their action and associated resistance mechanisms.

Polikanov et al. provide a detailed review of ribosome-targeting peptide antibiotics, with specific emphasis on each drug's interaction with either the small (30S) or large (50S) ribosome subunit and mechanism of action. Accumulating information on these antibiotics, including high-resolution ribosome-drug structures, offers opportunities to develop improved, next generation antibiotics with enhanced activity and, through modification of regions dispensable for ribosome inactivation, improvements in other properties such as uptake/retention or reduced toxicity.

Markley and Wencewicz describe the known mechanisms of resistance to tetracyclines, drugs that have been in clinical use for over 60 years. Resistance via efflux, ribosome modification, and the action of ribosome protection proteins are well-established, but their effects have been successfully countered through the design of more recent generations of tetracyclines such as tigecycline. However, these drugs are also now threatened by the emergence of the tetracycline-inactivating enzymes, which are the main focus of this review. Similarly, Golkar et al. describe the chemical structures, mechanisms of action, and resistance for a second major class of drugs, the macrolides, which bind in the peptide exit tunnel. Like tetracyclines, macrolides are subject to resistance via efflux, ribosome modification or mutation, and protection proteins. Additionally, their efficacy is also threatened by macrolide modifying phosphotransferase and esterase enzymes, the structures, and activities of which are the major focus of this comprehensive review.

## Sulfonamides and β-lactamases: Resistance and Frontiers in Drug Development

Sulfa drugs (sulfonamides) were first introduced in the 1930s and have a long history of efficacy against bacterial disease. These drugs inhibit bacterial dihydropteroate synthase (DHPS) by mimicking one of its substrates, *para*-aminobenzoic acid (PABA). Mutations in DHPS cause resistance to sulfonamides but their mechanism is often unknown. Griffith et al. identify five mutations of DHPS associated with sulfonamide resistance in *Staphylococcus aureus* and investigate their impacts on strain susceptibility and fitness, and enzyme kinetics. Three of the mutations contribute to resistance by sterically blocking the outer ring moiety of sulfonamides, whereas the other two increase fitness of the strain. The work reveals a critical weakness of sulfonamides with implications for drug design: resistance mutations target the part of the antimicrobial that is most important for its efficacy.

Discussion of antimicrobial resistance would not be complete without mention of β-lactamases, a common mechanism of resistance in bacteria, including the ESKAPE pathogens. These enzymes hydrolyze β-lactam antibiotics before they reach their molecular targets, the so-called penicillin-binding proteins. In his review, Palzkill explains the molecular basis for the differing specificities of three important groups of Class A β-lactamases (the TEM, CTX-M, and KPC enzymes) for oxyamino-cephalosporins. He highlights mutations that increase the conformational heterogeneity within the active sites of these enzymes to accommodate cephalosporins and the existence of global suppressor mutations elsewhere in the protein to compensate for loss of stability. Finally, in their review, van den Akker and Bonomo describe extensive efforts by a number of groups to develop β-lactamase inhibitors, including five approved for clinical use. They emphasize the success of strategies that exploit specific aspects of enzyme mechanism in the design of these critical antimicrobial agents.

## Author Contributions

All authors listed have made a substantial, direct and intellectual contribution to the work, and approved it for publication.

### Conflict of Interest Statement

The authors declare that the research was conducted in the absence of any commercial or financial relationships that could be construed as a potential conflict of interest.

## References

[B1] Vargiu A. V. Pos K. M. Poole K. Nikaido H. (2016). Editorial: bad bugs in the XXIst century: resistance mediated by multi-drug efflux pumps in gram-negative bacteria. Front. Microbiol. 7:833. 10.3389/fmicb.2016.00833 27303401 PMC4885826

